# Lynx1 Prevents Long-Term Potentiation Blockade and Reduction of Neuromodulator Expression Caused by Aβ1-42 and JNK Activation 

**Published:** 2018

**Authors:** M. L. Bychkov, N. A. Vasilyeva, M. A. Shulepko, P. M. Balaban, M. P. Kirpichnikov, E. N. Lyukmanova

**Affiliations:** Lomonosov Moscow State University, Leninskie Gori 1, Moscow, 119234, Russia; Shemyakin-Ovchinnikov Institute of Bioorganic Chemistry, Russian Academy of Sciences, Miklucho- Maklaya Str., 16/10, Moscow, 117997, Russia; Institute of Higher Nervous Activity and Neurophysiology, Russian Academy of Sciences, Butlerova Str., 5A, Moscow, 117485, Russia

**Keywords:** nicotinic acetylcholine receptor, cognitive impairment, Alzheimer disease, β-amyloid peptide, Ly6/uPAR

## Abstract

Nicotinic acetylcholine receptors (nAChRs) are ligand-gated ion channels. Many neurodegenerative diseases are accompanied by cognitive impairment associated with the dysfunction of nAChRs. The human membrane-tethered prototoxin Lynx1 modulates nAChR function in the brain areas responsible for learning and memory. In this study, we have demonstrated for the first time that the β-amyloid peptide Aβ_1-42_ decreases *Lynx1 *mRNA expression in rat primary cortical neurons, and that this decrease is associated with the activation of c-Jun N-terminal kinase (JNK). In addition, we have demonstrated that the *Lynx1 *expression decrease, as well as the blockade of the long-term potentiation underlying learning and memory, caused by Aβ_1-42_, may be prevented by incubation with a water-soluble Lynx1 analogue. Our findings suggest that the water-soluble Lynx1 analogue may be a promising agent for the improvement of cognitive deficits in neurodegenerative diseases.

## INTRODUCTION

Many neurodegenerative diseases, such as Alzheimer’s disease (AD), are characterized by impaired cognitive processes associated with the dysfunction of nicotinic acetylcholine receptors (nAChRs) [1]. In AD, oligomers of the β-amyloid peptide (Aβ) form plaques and the most toxic Aβ is Aβ_1-42_ [1]. Aβ_1-42_ in a 200 nM concentration inhibits α7-nAChR, the most common nicotinic cholinergic receptor in the brain; and the interaction between Aβ and the receptor leads to internalization of the latter in AD [1]. In addition, Aβ inhibits the long-term potentiation (LTP) [2] that is a generally established model for the plasticity processes underlying memory and learning [3].

Previously, we demonstrated that a water-soluble variant of the human protein Lynx1 (ws-Lynx1) [4], which modulates the α7-nAChR function in the brain [5], competes with Aβ1-42 for binding to α7-nAChR [6]. Pre-incubation of mouse cortical neurons with ws- Lynx1 was shown to reduce the cytotoxic effect of Aβ_1-42_ [6]. In addition, Western blot analysis revealed a reduced Lynx1 expression in the cortex of AD modeling transgenic mice (3×Tg-AD) compared to wild-type mice [6]. Based on these facts, we argue that Lynx1 plays an important role in AD, and that the accumulation of Aβ_1-42_ down-regulates the expression of this neuromodulator in the brain and disturbs the Aβ_1-42_/ Lynx1 balance, causing α7-nAChR dysfunction. We studied the effect of Aβ_1-42_ on *Lynx1 *gene expression in rat primary cortical and hippocampal neurons and evaluated the effect of ws-Lynx1 and Aβ1-42 on LTP in mouse hippocampal slices.

## EXPERIMENTAL

A primary neuron culture was prepared from the cortex and hippocampus of newborn Wistar rats according to the previously described procedure [7]. On the 14^th^ day, the neuron culture was supplemented with either Aβ_1-42_ (1 or 5 μM, Biopeptide Co) oligomerized according to the previously described protocol [8], or 5 μM Aβ_42-1_ (reverse peptide used as a negative control; Biopeptide Co), or 10 μM ws-Lynx1 (prepared according to [4]), or a mixture (5 μM Aβ_1-42_ + 10 μM ws-Lynx1), or 2.5 μM SP600125 (Tocris), or a mixture (5 μM Aβ_1-42_ + 2.5 μM SP600125) and incubated for an additional 24 h. For JNK knockdown, on the 10^th^ day cortical neurons were transfected with *JNK1 *and *JNK2 *small interfering RNAs (siRNAs) or with the control siRNA (*Table 1*). After that, the neurons were incubated for 72 h, then they were supplemented with 5 μM Aβ_1-42_ and incubated for another 24 h.

**Table 1:** The small interfering RNAs used in the study

Gene	Interfering RNA sequence
Control	UUCUCCGAACGUGUCACGUTT
	ACGUGACACGUUCGGAGAATT
JNK1	GGCAUGGGCUAUAAAGAAATT
	UUUCUUUGUAGCCCAUGCCTT
JNK2	GCCAGAGACUUAUUAUCAATT
	UUGAUAAUAAGUCUCUGGCTT

Then, the total mRNA was isolated using an ExtractRNA reagent (Evrogen). The mRNA was treated with DNase I (Thermo Fisher Scientific, USA), and cDNA was then synthesized using a MMLV RT kit (Evrogen). Real-time PCR was carried out using a 5x mixture of qPCRmix-HS SYBR + HighROX (Evrogen); the list of primers is given in *Table 2*. The data were analyzed using the LinReg 2017.0 software. The mRNA level was normalized to the β-actin values.

Transversal hippocampal slices from eight-month-old C57BL/6 mice were perfused with an artificial cerebrospinal fluid (ACSF) (124 mM NaCl, 3 mM KCl, 2.5 mM CaCl_2_, 1.3 mM MgCl_2_, 26 mM NaHCO_3_, 1.27 mM NaH_2_PO_4_, and 10 mM *D*-glucose, pH 7.4), continuously saturated with carbogen (95% O_2_ + 5% CO_2_) at 34 °C for 1 h. Then, a portion of the slices was perfused with ACSF containing 200 nM Aβ_1-42_ and the other was perfused with ACSF containing 200 nM Aβ_1-42_ + 2 μM ws- Lynx1 for 1 h. Control slices were perfused with ACSF without Aβ_1-42_ and ws-Lynx1. Field excitatory postsynaptic potentials (fEPSPs) were recorded using a SliceMaster system (Scientifica, UK) at 32°C. A recording electrode (1-3 MΩ) filled with ACSF was positioned within hippocampal CA1 stratum radiatum. Synaptic responses were evoked by paired-pulse stimulation of Schaffer collaterals in the CA3 stratum radiatum area by using a bipolar electrode. A 50-ms interpulse interval was used, unless stated otherwise. The simulations were repeated at 0.033 Hz. Stimulus intensity was adjusted to elicit 40 % of maximal fEPSP amplitude.

After 20 minutes of recording test responses, a high-frequency stimulation (HFS) protocol was used to induce LTP: 10 trains with a frequency of 100 Hz (five stimuli per train) with an intertrain interval of 200 ms, four sessions with an interval of 30 s. After LTP induction, fEPSPs were recorded for 1.5 h. The obtained data were recorded, filtered, and analyzed using the Spike2 software (Cambridge Electronic Design Limited, UK) and SigmaPlot 11.0 (Systat Software Inc., USA). The post-tetanic tangent of the fEPSP slope was normalized to the mean slope of all fEPSPs recorded 20 min before LTP induction.

The statistical analysis of the LTP data and the data on the effect of Aβ1-42, ws-Lynx1, SP600125, and siRNA on gene expression in primary neurons was performed using the GraphPad Prism 6.0 (GraphPad Software Inc.) software. A value of *p * < 0.05 was considered statistically significant. All experiments were performed in accordance with the guidelines set forth by the European Communities Council Directive of November 24, 1986 (86/609/EEC) and were approved by the ethical committees of the Shemyakin-Ovchinnikov Institute and Institute of Higher Nervous Activity and Neurophysiology, Russian Academy of Sciences.

## RESULTS AND DISCUSSION

**Aβ1-42–induced decrease in *Lynx1* expression in neurons is associated with JNK activation**

**Table 2:** Primers used in the study

Gene	Forward primer	Reverse primer	Length, bp
β-actin	TCATGTTTGAGACCTTCAACAC	GTCTTTGCGGATGTCCACG	250
Lynx1	ACCACTCGAACTTACTTCACC	ATCGTACACGGTCTCAAAGC	81
α7-nAChR	TGCACGTGTCCCTGCAAGGC	GTACACGGTGAGCGGCTGCG	112

**Fig. 1:**
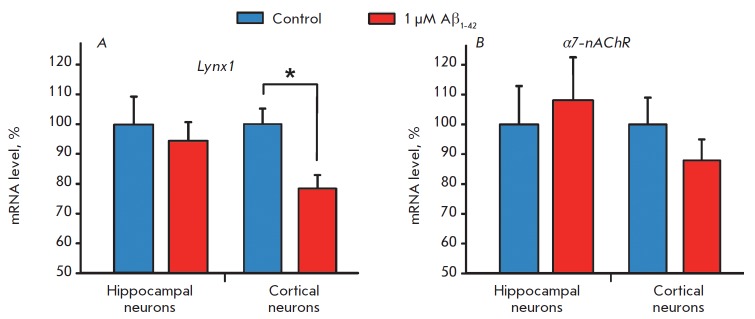
Effect of Aβ_1-42_ on *Lynx1 *(A) and *α7-nAChR *(B) gene expression in primary cortical and hippocampal neurons. The data are presented as % of the control ± s.e.m. (n = 3). Data indicated by * (p < 0.05) significantly differ from each other, based on a two-sided t-test

**Fig. 2:**
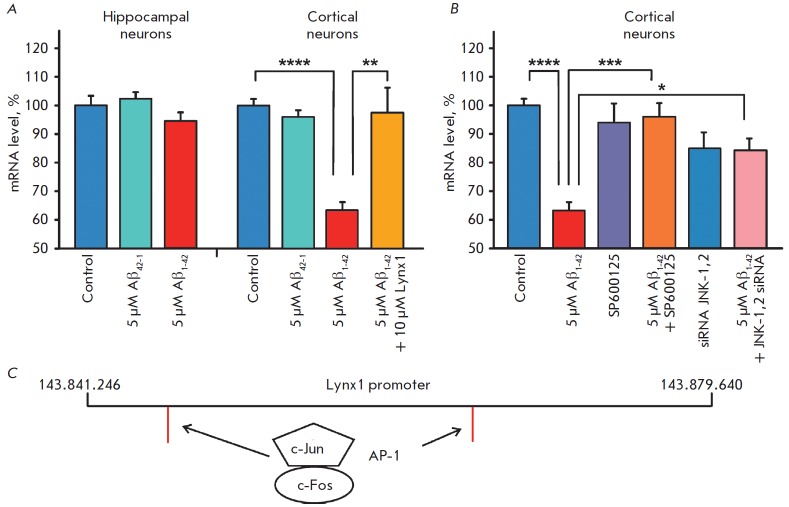
Ws-Lynx1 and JNK inhibition cancels the decrease in *Lynx1 *expression in a primary culture of cortical neurons treated with Aβ_1-42_. (*A*) Effect of Aβ_1-42_, Aβ_42-1_, and ws-Lynx1 on *Lynx1 *expression. (*B*) Effect of Aβ_1-42_, JNK inhibition by SP600125, and knockdown of the *JNK1 *and *JNK2 *genes on *Lynx1 *expression. The data are presented as % of the control ± s.e.m. (*n *= 4). Data indicated by * (*p * < 0.05), ** (*p * < 0.01), and **** (*p * < 0.0001) mean a statistically significant difference between groups according to the one-sided ANOVA test, followed by the Tukey’s/hoc test. (*C*) Schematic structure of the *Lynx1 *gene. Red lines denote the c-Jun and c-Fos binding sites.

To test the hypothesis of the effect of amyloid peptide on Lynx1 expression, we incubated primary neurons of the rat cortex and hippocampus with 1 μM of oligomeric Aβ_1-42_ and analyzed the Lynx1 mRNA level gomeric Aβ_1-42_ and analyzed the *Lynx1 *mRNA level (*Fig. 1A*). In hippocampal neurons, there was no significant decrease in neuromodulator expression, while a significant reduction in the *Lynx1 *mRNA level (up to 78.4 ± 4.4% of the control level) was observed in cortical neurons. This is consistent with a previously observed decrease in Lynx1 expression in the cortex of AD modeling mice [6]. In contrast, Aβ_1-42_ did not decrease the *α7-nAChR *mRNA level either in hippocampal or in cortical neurons (*Fig. 1B*). An increase in the Aβ_1-42_ concentration to 5 μM led to a further decrease in *Lynx1* gene expression in cortical neurons (up to 65.8 ± 4.9% of the control level, *Fig. 2A*).

Nicotine-induced activation of α7-nAChR can regulate gene transcription through CREB phosphorylation and the activation of MAP/ERK signaling pathways, which is accompanied by an increase in the expression level of the early response c-Fos transcription factor [9]. On the other hand, binding of oligomeric Aβ1-42 to α7-nAChR leads to the activation of c-Jun N-terminal kinase (JNK) [10], which plays a key role in the regulation of gene expression and other vital processes, including processing of the β-amyloid peptide precursor and formation of neurofibrillary tangles in AD [10]. In turn, JNK activation may lead to the inhibition of CREB transcription factor phosphorylation and, therefore, to a decrease in the expression level of the c-Fos transcription factor [11].

To elucidate whether the decreased *Lynx1 *expression level in cortical neurons incubated with oligomeric Aβ_1-42_ was associated with JNK activation, we incubated cortical neurons with Aβ_1-42_ and SP600125, a selective inhibitor of JNK1, JNK2, and JNK3 which is considered now as one of the potential drugs for AD treatment [10]. Indeed, co-incubation of neurons with Aβ_1-42_ and SP600125 prevented the decrease in *Lynx1 *expression, indicating a possible association of this decrease with JNK activation (*Fig. 2B*). To confirm the role of JNK in the regulation of *Lynx1 *transcription, we used knockdown of the *JNK1 *and *JNK2 *genes with small interfering RNAs. As expected, incubation of neurons with blocked *JNK1 *and *JNK2 *expression in the presence of Aβ_1-42_ led to a recovery of the *Lynx1 *mRNA expression level (*Fig. 2B*). In this case, transfection of the neuronal culture with control siRNA that did not inhibit gene transcription had no effect on the decrease in *Lynx1 *mRNA expression caused by Aβ_1-42_ (data not shown). Knockdown of *JNK1 *and *JNK2 *in the absence of Aβ_1-42_ did not cause significant changes in the *Lynx1 *expression level (*Fig. 2B*), which confirms the association of the amyloid peptide, JNK activation, and decreased neuromodulator transcription.

An analysis of the human *LYNX1 *gene promoter in the human genome browser (chr8: 143841246 – chr8: 143879640) and the mouse *Lynx1 *gene promoter (chr15: 74573409 – chr15: 74603409) revealed two potential binding sites for the AP-1 transcriptional complex formed by the c-Jun and c-Fos transcription factors (*Fig. 2C*). Aβ_1-42_-induced activation of JNK is simultaneously accompanied by c-Jun activation [10] and c-Fos down-regulation [11]. For that reason, a possible imbalance between c-Jun and c-Fos can cause a disruption in the AP-1 transcriptional complex formation and lead to the decrease in *Lynx1 *gene transcription. In accordance with this suggestion, incubation of cortical neurons together with ws-Lynx1 and Aβ_1-42_ was accompanied by recovery of the *Lynx1 *mRNA level (*Fig. 2B*). Apparently, ws-Lynx1 competes with Aβ_1-42_ for binding to α7-nAChR [6] and activates α7-nAChR in a nicotine-like manner, which leads to the c-Fos up-regulation [9] and recovery of *Lynx1 *transcription.

**Ws-Lynx1 prevents Aβ_1-42_–induced LTP blockade**

**Fig. 3:**
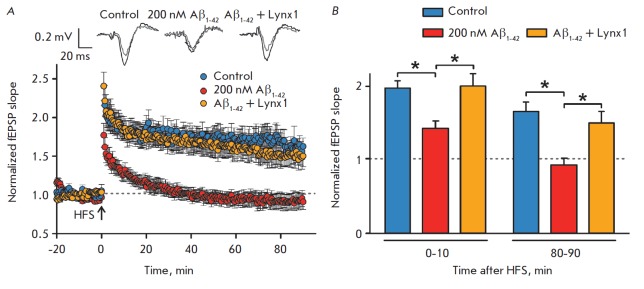
Ws-Lynx1 prevents Aβ_1-42_-induced LTP blockade in hippocampal slices.(*A*) Time course of changes in the fEPSP slope recorded in control hippocampal slices perfused with ACSF without Aβ_1-42_ (*n *= 8), ACSF containing 200 nM Aβ_1-42_ (*n *= 6), and ACSF containing 200 nM Aβ_1-42_ + 2 μM ws-Lynx1 (*n *= 5). (*B*) fEPSPs slopes averaged during 0–10 min and 80–90 min after HFS. * (*p* < 0.05) means a statistically significant difference between groups according to the one-way ANOVA test, followed by the Tukey’s/hoc test

Using SP600125, it was previously demonstrated that the LTP blockade observed during the incubation of hippocampal slices with oligomeric Aβ_1-42_ was associated with JNK activation [12]. To study the effect of Lynx1 on the recovery of the synaptic plasticity impaired by the interaction between oligomeric Aβ_1-42_ and α7-nAChR, as well as JNK activation, we investigated the influence of 200 nM Aβ_1-42_ on LTP in the surviving mouse’s hippocampal slices in the presence and absence of 2 μM ws-Lynx1. Pre-perfusion of the slices in a solution containing Aβ_1-42_ for 1 h led to a significant decrease in the post-tetanic fEPSP, noticeable in the first minutes after LTP induction. In this case, the fEPSP slope averaged over the first 10 minutes of recording decreased almost 1.5-fold compared to the control fEPSP slope (*Fig. 3B*). A significant decrease in the fEPSP slope caused by Aβ_1-42_ to the baseline fEPSP values was observed during the entire period after LTP induction.

However, incubation of hippocampal slices in a medium containing both Aβ_1-42_ and ws-Lynx1 restored the LTP level almost to the control values (*Fig. 3*). The mean fEPSP slope upon simultaneous application of Aβ_1-42_ and ws-Lynx1 was significantly higher than that of the fEPSP observed during incubation with Aβ_1-42_ alone and was not statistically different from the mean fEPSP slope in the control throughout the recording time after HFS (*Fig. 3B*). Therefore, ws-Lynx1 prevents the inhibitory effect of Aβ_1-42_ and facilitates the complete recovery of LTP.

## CONCLUSION

Hereby, the presence of oligomeric Aβ_1-42_ in the neuronal environment leads to a significant decrease in the expression of the Lynx1 neuromodulator that regulates α7-nAChR functioning in the brain. We have demonstrated for the first time that this decrease is associated with JNK activation and can be prevented by incubation with the water-soluble Lynx1 analogue. In addition, ws-Lynx1 is capable of correcting Aβ_1-42_–induced impairments of hippocampal synaptic plasticity, which underlies memory impairment and other cognitive dysfunctions in AD.

## Abbreviations

ACSF: artificial cerebrospinal fluid
AD: Alzheimer disease
HFS: high-frequency stimulation,
LTP: long-term potentiation
fEPSPs: field excitatory postsynaptic potentials
Aβ: β-amyloid peptide
nAChR: nicotinic acetylcholine receptor
siRNA: small interfering RNA
ws-Lynx1: water-soluble Lynx1
